# Single-lung ventilation technique in neonates undergoing thoracoscopic repair of esophageal atresia: a single-center retrospective cohort study

**DOI:** 10.3389/fsurg.2024.1446586

**Published:** 2024-11-19

**Authors:** Fan Zhang, Zhijian Zhou, Yingbei Liu, Xuan Wang

**Affiliations:** ^1^ Department of Anesthesiology, Children’s Hospital of Fudan University, Shanghai, China; ^2^ Department of Cardiothoracic Surgery, Children’s Hospital of Fudan University, Shanghai, China

**Keywords:** esophageal atresia, neonate, single-lung ventilation, thoracic surgery, minimal invasive surgery

## Abstract

**Background:**

Thoracoscopic repair is a common surgical procedure to treat esophageal atresia (EA). During thoracoscopic surgery, the single-lung ventilation (SLV) technique is used to collapse one of the lungs to obtain a better surgical view. However, SLV is associated with risks in neonates. This study aimed to assess the perioperative benefits and risks of SLV in neonates who underwent thoracoscopic EA repair.

**Methods:**

This single-center retrospective cohort study included all neonates who underwent thoracoscopic repair of EA at the Children's Hospital of Fudan University between January 1, 2016 and December 31, 2021. Neonates were assigned to SLV (Group S) or dual-lung ventilation (DLV, Group D) groups depending on the technique used intraoperatively. The intraoperative and postoperative information of the two groups were compared.

**Results:**

A total of 70 neonates were included in this study. Twenty-nine neonates were assigned to Group S and forty-one to Group D. No intraoperative adverse events were observed in either group. The surgery time of Group S was significantly shorter than that of Group D (81 ± 23 and 99 ± 29 min, respectively, *P* = 0.004). In contrast, the anesthetic preparation time of Group S was significantly longer than that of Group D (54 ± 22 and 44 ± 16 min, respectively, *P* = 0.030). The frequency of postoperative adverse events in Group S was similar to that of Group D (31.03% and 40.54%, respectively, *P* = 0.453).

**Conclusion:**

SLV was associated with a reduced surgery time for thoracoscopic repair of EA and longer anesthetic preparation time compared to DLV. The SLV was as safe as the DLV with potential advantages in thoracoscopic EA repair.

## 1 Introduction

Congenital esophageal atresia (EA) is the most common severe esophageal anomaly. Thoracoscopic repair is a surgical technique widely used to treat EA (1). Thoracoscopic repair is as safe as conventional open repair (2) with a lower frequency and severity of thoracic musculoskeletal deformities (3, 4). However, the lung in the surgical site needs to be collapsed during the thoracoscopic surgery. Although the dual-lung ventilation (DLV) technique can be used intraoperatively with the help of artificial pneumothorax using carbon dioxide, it may not always completely collapse the lung. The simple increase in the pressure of the artificial pneumothorax can affect the hemodynamic and respiratory functions of neonates. The single-lung ventilation (SLV) technique, routinely used in adult thoracoscopic surgeries, can promote more effective lung collapse, creating better conditions for surgeons (5). The SLV has been successfully used in both term (6) and preterm (7) infants with EA and safely performed without respiratory compromise (8). Nevertheless, the use of SLV in neonates with EA is challenging and presents risks due to the small diameter of their trachea; thus, intubating and ventilating neonates using SLV is difficult. Moreover, special physiology and pathophysiology conditions, such as lower functional residual capacity, higher oxygen consumption, and preoperative pneumonitis, may lead to intraoperative hypoxia when ventilating only one lung (9). Although assessing the benefits and risks of SLV in neonates undergoing thoracoscopic repair of EA is important, data on the efficacy and safety of SLV in these patients is currently limited. Therefore, this study analyzed neonates with EA who underwent thoracoscopic repair of EA at a university-affiliated children's hospital between January 1, 2016 and December 31, 2021 to determine whether SLV can be safely used in neonates with EA and benefit them perioperatively.

## 2 Methods

This single-center retrospective cohort study was approved by the Ethics Committee of our hospital [no. (2022) 248]. Written informed consent was obtained from the guardians of all neonates. This study analyzed neonates with EA who underwent thoracoscopic repair of EA at a university-affiliated children's hospital between January 1, 2016 and December 31, 2021.

### 2.1 Inclusion criteria

The inclusion criteria were as follows: (1) congenital EA as a preoperative diagnosis, (2) age ≤ 30 days, (3) planned thoracoscopic EA repair, and (4) no previous surgical history.

### 2.2 Exclusion criteria

The exclusion criteria were as follows: (1) an intraoperative diagnosis inconsistent with the preoperative diagnosis and (2) extensive missing information.

### 2.3 Data acquisition

Data were collected from the anesthetic recording and the electronic inpatient medical record systems of our hospital. The data in the anesthetic recording system were collected automatically, and vital signs were captured every 5 min during anesthesia. All data were collected independently by two investigators. If the data collected by each investigator presented differences, the two investigators jointly queried the medical record system to determine the final data.

Data of demographic information, whether the neonate was intubated and mechanically ventilated preoperatively, the type of EA, preoperative pulse oxygen saturation (SpO_2_), all congenital anomalies, preoperative blood gas test results, attending anesthesiologists, whether the surgery was elective or emergency, anesthetic preparation time, surgery time, total surgical time, whether the SLV technique was used, lowest SpO_2_ and highest end-tidal carbon dioxide (ETCO_2_) values during surgery, all perioperative adverse events, postoperative mechanical ventilation time, postoperative neonatal intensive care unit (NICU) time, postoperative hospital stay length, and mortality during hospital stay from the anesthetic recording and inpatient medical recording systems were also collected.

Anesthetic preparation time was defined as the time from the pre-induction assessment to the completion of all anesthetic preparation before surgery. Surgery time was defined as the time from the start of the incision to the completion of suturing. Total surgical time was defined as the time from the patient entering the operating room to the time when the patient left the operating room.

All neonates were assigned to either the SLV group (Group S) or the DLV group (Group D) according to whether SLV was used intraoperatively. The decision whether SLV was used intraoperatively was made by the anesthesiologist responsible for the surgery.

### 2.4 Statistical analysis

SPSS 26 (version R26.0.0.0, 64-bit; IBM Corporation, Armonk, NY, USA) was used for statistical analyses. Pearson's chi-squared or Fisher's exact tests were used to assess categorical data. The data of age and gestational age were expressed as median (interquartile range) and analyzed using the Mann–Whitney *U* test. The other measurement data were expressed as mean ± standard deviation values and a *t*-test or non-parametric test was used according to whether they were consistent with the normal distribution. *P* < 0.05 was considered statistically significant.

## 3 Results

**Figure 1:**
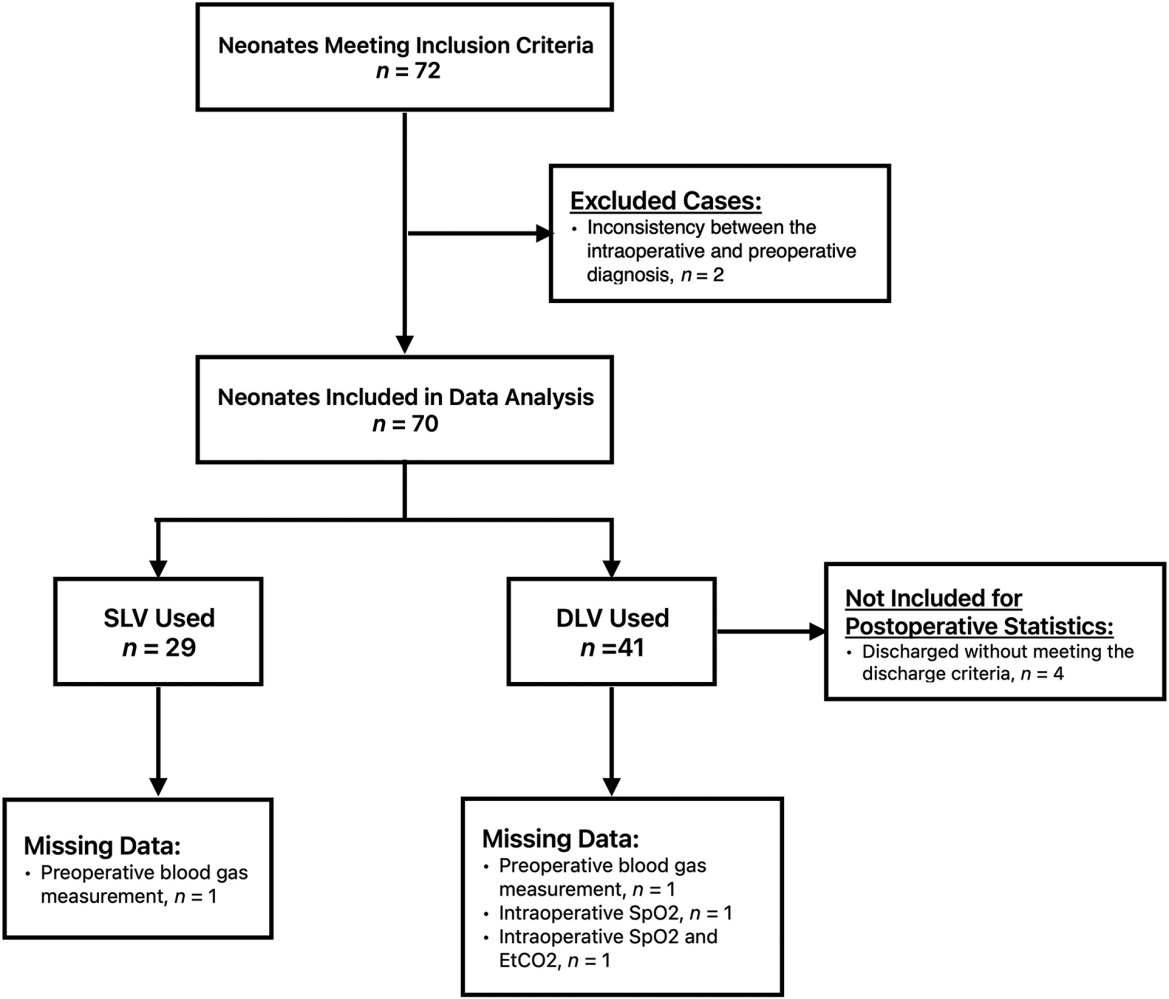
Study flow chart.

A total of 72 neonates were initially eligible for this study, of which two were eventually excluded due to inconsistencies between the intraoperative and preoperative diagnoses. Following the exclusion criteria, 70 patients were included in the study. Twenty-nine neonates were assigned to Group S and forty-one patients were assigned to Group D (Figure 1). Both groups consisted of neonates who underwent surgery within the period investigated in this study. One neonate in Group D was converted to thoracotomy intraoperatively because the esophageal anastomotic stoma was under tension. All neonates had type C EA. The baseline characteristics and preoperative capillary blood gas test results of the two groups are shown in Table 1. One preoperative blood gas measurement in Group S and two measurements in Group D were not recorded. The preoperative SpO_2_ of one neonate in Group S was not recorded in the medical recording system. Therefore, these data were not included in the statistical analyses.

**Table 1:** Baseline characteristics of two groups.

	Group S (*n* = 29)	Group D (*n* = 41)	*P*
Sex (M/F)	14/15	19/22	0.873
Preterm (Y/N）	9/20	10/31	0.538
Gestational Age (weeks)	38 (36^+3^, 39^+5^)	38^+4^ (37, 39^+2^)	0.929
(Range)	(32^+5^–41^+1^)	(32^+3^–43)	
Age (days)	3 (2, 4)	3 (2,4)	0.440
(Range)	(0–11)	(1–16)	
Weight (kg)	2.57 ± 0.65	2.55 ± 0.50	0.919
(Range)	(1.60–3.80)	(1.30–3.60)	
Preoperative SpO_2_ (%)	95 ± 2*	95 ± 2	0.908
Intubated (Y/N)	3/26	6/35	0.726
Associated Anomalies			
Cardiac	3	8	0.342
Anorectal	3	3	0.686
Renal	0	3	0.261
Limb	0	4	0.136
Vertebral	0	1	1.000
Laryngeal	1	2	1.000
Charge syndrome	1	0	0.414
Preoperative blood gas			
pH	7.383 ± 0.072*	7.382 ± 0.061**	0.965
PO_2_ (mmHg)	79.0 ± 56.0*	61.0 ± 32.4**	0.134
PCO_2_ (mmHg)	39.8 ± 8.1*	40.9 ± 7.5**	0.575
Hb (g/dl)	16.4 ± 2.4*	16.4 ± 3.5**	0.931
BE	−1.9 ± 3.9*	−1.0 ± 1.1**	0.296
Lactic acid (mmol/L)	2.3 ± 1.1*	2.2 ± 1.2**	0.818

* *n* = 28.

** *n* = 40.

### 3.1 Intraoperative management and outcomes

All patients underwent anesthesia and surgery without any adverse intraoperative events. None of the patients were administered pre-medication. The blood loss in each case was ≤5 ml during the surgery. In Group S, as recorded in the anesthetic records and according to the department routine, all neonates underwent fibro-bronchoscopy examination with spontaneous breathing after inhalation induction with sevoflurane to determine the location of the tracheoesophageal fistula (TEF) and clean the airway. All neonates in Group S were intubated through the left bronchus. A 3.0 mm inner diameter (ID) uncuffed endotracheal tube [4.2 mm outer diameter (OD)] was used to intubate the left mainstem bronchus. If intubating the left bronchus was difficult, a 2.5 mm ID cuffless endotracheal tube (3.5 mm OD) was used. The common way to insert an endotracheal tube into the left mainstem bronchus was to rotate the bevel of the endotracheal tube 180° and turn the head of the patient to the right. In some cases, a 24 Ga guidewire was inserted into the left bronchus under fibro-bronchoscopy, and then an endotracheal tube was advanced over the guidewire into the bronchus. In this study, the neonates under 2.5 kg were intubated with a 2.5 mm endotracheal tube, and the neonates over 3.0 kg were intubated with a 3.0 mm endotracheal tube. All neonates were mechanically ventilated during the operation. In Group D, the neonates were anesthetized with sevoflurane and intubated under spontaneous breathing with an uncuffed tube. In addition, the patients in Group D were ventilated either spontaneously or mechanically before TEF ligation and mechanically ventilated after TEF ligation. Patients in both groups were placed in a 3/4 prone position, and artificial pneumothorax was established using CO_2_ at a pressure of 6 mmHg and a flow rate of 4–5 L/min.

The intraoperative information of both groups is shown in Table 2. The surgical time of Group S was significantly shorter than that of Group D (81 ± 23 and 99 ± 29 min, respectively, *P* = 0.004). In contrast, the anesthetic preparation time of Group S was significantly longer than that of Group D (54 ± 22 and 44 ± 16 min, respectively, *P* = 0.030). The total surgical time of the two groups was similar (139 ± 32 and 150 ± 40 min, *P* = 0.209). In the Group S, 11 neonates (39.29%) presented the lowest intraoperative SpO_2_ values of <90%, including three (10.71%) with values of <80%. In Group D, the intraoperative SpO_2_ of one patient was not recorded and 12 neonates (30%) presented the lowest intraoperative SpO_2_ values of <90%, including three (7.5%) with values of <80%. However, none of the patients were desaturated for >5 min.

**Table 2:** Intraoperative information of two groups.

	Group S	Group D	Difference	95% CI	*P*
	(*n* = 29)	(*n* = 41)			
Anesthetic preparation time (min)	54 ± 22	44 ± 17	10 ± 5	1, 20	0.031
Surgery time (min)	81 ± 23	99 ± 29	−18 ± 6	−31, −6	0.004
Total surgical time (min)	139 ± 32	150 ± 40	−11 ± 9	−26, 7	0.209
SpO_2_ < 90%	11 (39.29%)*	12 (31.70%)**			0.729
Lowest SpO_2_ (%)	89 ± 7*	90 ± 10**			0.650
Highest EtCO_2_ (mmHg)*	56 ± 15*	65 ± 13			0.010

* *n* = 28.

** *n* = 40.

### 3.2 Postoperative management and outcomes

All the patients in both groups were transferred to the NICU with a tracheal tube by the NICU transfer team. Following the request of their parents, four neonates in Group D were discharged without meeting the discharge criteria. Therefore, these four cases in Group D were not included in the postoperative statistics.

Postoperative mechanical ventilation time, NICU stay length, and postoperative hospital stay length are shown in Table 3. Nine patients in Group S presented with postoperative adverse events (Table 4). The neonate with subglottic stenosis diagnosed by rigid bronchoscopy was treated with tracheal balloon dilatation after correction and was discharged, meeting the discharge criteria. Five neonates with anastomotic strictures were treated with esophageal dilatation. The other three neonates with adverse events were discharged without any unplanned reoperations. In contrast, in Group D 15 of 37 patients presented postoperative adverse events (Table 5). In Group S, eight unplanned reoperations were performed in seven patients, which means that one patient in this group was subjected to two unplanned reoperations. In contrast, in Group D 15 unplanned reoperations were performed in 12 patients (one patient underwent four unplanned reoperations). In Group S, seven patients underwent gastroscopic esophageal dilatation due to anastomotic stenosis, and one underwent tracheal balloon dilatation as described above. In Group D, ten neonates underwent gastroscopic esophageal dilatation because of anastomotic stenosis, including one neonate who underwent one pass of gastroscopic balloon esophageal dilatation and three passes of gastroscopic esophageal dilatation postoperatively. One neonate underwent gastroscopy and bronchoscopy to diagnose TEF recurrence. One neonate underwent a gastrostomy owing to anastomotic leakages. None of the neonates in both groups died perioperatively.

**Table 3:** Postoperative information of two groups.

	Group S	Group D	Difference	95% CI	*P*
	(*n* = 29)	(*n* = 37)			
Mechanical ventilation time (days)	4.6 ± 1.7	4.8 ± 5.3	−0.2 ± 0.9	−2.1, 1.7	0.826
NICU time (days)	16 ± 13	16 ± 18	0.5 ± 4	−8, 8	0.911
Hospital stay (days)	28 ± 11	33 ± 21	−5 ± 4	−13, 3	0.252
Adverse events	9 (31.03%)	15 (40.54%)			0.453
Respiratory	3 (10.34%)	5 (13.51%)			
Surgical	6 (20.69%)	13 (35.14%)			
Unplanned reoperations	7 (24.14%)	11 (32.43%)			0.782

**Table 4:** The information of postoperative adverse events and treatments of group S.

No.	Gestational age (days)	Weight (kg)	Associated anomalies	Adverse event	Treatments
1	239	1.60	Rectal navicular fossa fistula	Unilateral vocal cord paralysis and AS	Esophageal dilatation
2	241	1.60	Type I laryngeal cleft, *FBN2* gene mutation	Subglottic stenosis and AS	Tracheal balloon dilatation and esophageal dilatation
3	236	1.70	None	AS	Esophageal dilatation
4	232	1.80	None	Pleural effusion	Chest drainage
5	255	2.00	None	Stenosis of the middle and low trachea	Observation
6	280	2.40	ASD	AS	Esophageal dilatation
7	261	2.50	None	AS	Esophageal dilatation
8	285	2.80	None	AS	Esophageal dilatation
9	288	3.40	None	AS	Esophageal dilatation

ASD, atrial septal defect; AS, anastomotic stricture.

**Table 5:** The information of postoperative adverse events and treatments of group D.

No.	Gestational age (days)	Weight (kg)	Associated anomalies	Adverse event	Treatments
1	227	1.50	Imperforate anus, ASD	Pneumothorax and AS	Esophageal dilatation for 3 times
2	244	1.80	None	AS	Esophageal dilatation
3	251	1.92	None	AS	Esophageal dilatation
4	234	2.00	None	Hypoxemia	Reintubation, return to NICU
5	267	2.60	Polycystic kidney, polydactyly	AS	Esophageal dilatation
6	259	2.63	None	Hypoxemia and AS	Return to NICU, esophageal dilatation
7	272	2.70	None	AS	Esophageal dilatation
8	280	2.70	ASD, laryngeal cartilage dysplasia	AS	Esophageal dilatation
9	271	2.80	None	AS	Esophageal dilatation
10	265	2.83	ASD, VSD	Hypoxemia	Reintubation, return to NICU
11	282	2.85	None	Pulmonary atelectasis and recurrence of TEF	Bronchoscopy examination and gastroscopy
12	289	2.97	None	Unilateral vocal cord paralysis	Observation
13	283	3.00	VSD	AS	Esophageal dilatation
14	277	3.13	None	AS	Esophageal dilatation
15	279	3.36	None	Anastomotic leakage	Gastrostomy

ASD, atrial septal defect; VSD, ventricular septal defect; TEF, tracheoesophageal fistula; AS, anastomotic stricture.

## 4 Discussion

This retrospective study found that SLV was associated with a reduction in surgery time and an increase in anesthetic preparation time during thoracoscopic EA repair, which is similar to data from thoracoscopic esophageal surgeries in adults. Lin et al. found that the SLV technique reduces the time of thoracoscopic esophageal surgery compared to artificial pneumothorax and DLV (10), probably due to the better exposure of the surgical site and reduced interruption from the collapsed lung when using the SLV (11). Few studies exploring the SLV technique in neonates with EA undergoing thoracoscopic repair are currently available due to the low incidence of EA and difficulties in enacting the SLV in neonates. Although the experience of surgeons can critically affect the surgical time (12), this factor may not be the reason for the results of this study since all these surgeries analyzed were performed by the same surgeon at our institution, and he possesses a rich experience.

It was not surprising that the anesthetic preparation time of Group S was significantly longer than that of Group D. The surgical site in our study was always on the right, and it is well-known that inserting an endotracheal tube into the left bronchus of the lung is difficult, which might lead to two or more attempts to establish SLV and prolong the anesthetic preparation time. Although the anesthetic preparation time was longer in Group S, the incidence of respiratory adverse events in the two groups was similar. One neonate was diagnosed with subglottic stenosis using bronchoscopy. This neonate weighed only 1.6 kg and had an *FBN2* mutation. The incidence of subglottic stenosis after intubation in neonates varies from 0.3%–11% (13), while the incidence of severe subglottic stenosis where surgery is needed is 0.93% (14). It is unclear whether SLV increases the risk of subglottic stenosis owing to the low incidence of postintubation subglottic stenosis and small number of cases in this study. Furthermore, Yin et al. found that *FBN2* is critically important in tracheal formation and is associated with tracheomalacia (15), which may be associated with subglottic stenosis of the neonate. Moreover, persistent intraoperative desaturation (SpO_2_ < 90% and lasting >5 min) was not observed in this study.

The highest ETCO_2_ of Group D was higher than that of Group S, which was also found in the minimally invasive esophagectomy in adults (10). One reason was the insufficient ventilation in Group D. The peak pressure of mechanical ventilation was limited in DLV because the high peak pressure could prevent the lung from adequately collapsing and worse the visualization of thoracoscopy.

This study had some limitations. As this was a retrospective study, the actual perioperative management protocols could not be strictly controlled and there might be slight differences. However, the same surgical team and anesthetic routine reduced the bias. Some perioperative events may not have been recorded due to deviations. In addition, this was a single-center study; thus, the number of cases available was limited and no neonates with severe associated anomalies were included in the study. As a result, the care of neonates with severe associated anomalies should be personalized and fully discussed to obtain the best outcomes. Moreover, our results were limited to patients with type C EA because none of these patients had other types of EA. Further randomized controlled trials are warranted to confirm the association between SLV and reduced surgery time in neonates with EA.

## Conclusion

In this study, the SLV technique was associated with a reduced surgery time and longer anesthetic preparation time compared to DLV. In addition, the SLV was as safe as the DLV. Therefore, the SLV technique is potentially advantageous in thoracoscopic EA repair.

## Data Availability

The raw data supporting the conclusions of this article will be made available by the authors, without undue reservation.
